# Sparse and Specific Coding during Information Transmission between Co-cultured Dentate Gyrus and CA3 Hippocampal Networks

**DOI:** 10.3389/fncir.2017.00013

**Published:** 2017-03-06

**Authors:** Daniele Poli, Srikanth Thiagarajan, Thomas B. DeMarse, Bruce C. Wheeler, Gregory J. Brewer

**Affiliations:** ^1^Department of Biomedical Engineering, University of CaliforniaIrvine, CA, USA; ^2^Department of Neurology, University of North CarolinaChapel Hill, NC, USA; ^3^Department of Biomedical Engineering, University of FloridaGainesville, FL, USA; ^4^Department of Bioengineering, University of CaliforniaSan Diego, CA, USA; ^5^Memory Impairments and Neurological Disorders (MIND) Institute, University of CaliforniaIrvine, CA, USA

**Keywords:** hippocampus, CA3, DG, network, micro-electrode array, neural code, decoding, machine learning

## Abstract

To better understand encoding and decoding of stimulus information in two specific hippocampal sub-regions, we isolated and co-cultured rat primary dentate gyrus (DG) and CA3 neurons within a two-chamber device with axonal connectivity via micro-tunnels. We tested the hypothesis that, in these engineered networks, decoding performance of stimulus site information would be more accurate when stimuli and information flow occur in anatomically correct feed-forward DG to CA3 vs. CA3 back to DG. In particular, we characterized the neural code of these sub-regions by measuring sparseness and uniqueness of the responses evoked by specific paired-pulse stimuli. We used the evoked responses in CA3 to decode the stimulation sites in DG (and vice-versa) by means of learning algorithms for classification (support vector machine, SVM). The device was placed over an 8 × 8 grid of extracellular electrodes (micro-electrode array, MEA) in order to provide a platform for monitoring development, self-organization, and improved access to stimulation and recording at multiple sites. The micro-tunnels were designed with dimensions 3 × 10 × 400 μm allowing axonal growth but not migration of cell bodies and long enough to exclude traversal by dendrites. Paired-pulse stimulation (inter-pulse interval 50 ms) was applied at 22 different sites and repeated 25 times in each chamber for each sub-region to evoke time-locked activity. DG-DG and CA3-CA3 networks were used as controls. Stimulation in DG drove signals through the axons in the tunnels to activate a relatively small set of specific electrodes in CA3 (sparse code). CA3-CA3 and DG-DG controls were less sparse in coding than CA3 in DG-CA3 networks. Using all target electrodes with the three highest spike rates (14%), the evoked responses in CA3 specified each stimulation site in DG with optimum uniqueness of 64%. Finally, by SVM learning, these evoked responses in CA3 correctly decoded the stimulation sites in DG for 43% of the trials, significantly higher than the reverse, i.e., how well-recording in DG could predict the stimulation site in CA3. In conclusion, our co-cultured model for the *in vivo* DG-CA3 hippocampal network showed sparse and specific responses in CA3, selectively evoked by each stimulation site in DG.

## Introduction

Understanding neural coding in the brain has proceeded with difficulty due to limitations on accessibility as well as conceptual precepts. After establishing the first principle of neural coding as the temporal pattern of the spikes rather than their shape (Adrian, 1928), it was easier to distinguish and classify different coding schemes for learning and memory. While the concept of memory as a linear connection of propagated activity persisted for decades, missing elements of intermediate-stage modulation, and selectivity persisted. The conceptual paradigm shift to memory as cell assemblies of temporally activated neurons introduced both conditional and coincident activation of small networks (Hebb, 1949). Identification of the layered structures in the hippocampal formation as the seat of cognitive learning and memory led Marr (1971) to propose constraints on computational models of hippocampal function in memory storage, introducing the efficiency of vast arrays of permuted sparse representations. Yet access to these arrays of sparse activity has remained limited both *in vivo* and even in hippocampal slices due extremes of downstream unresponsiveness to single neuron activation and widespread responses to stimulation of bundles of axon fiber tracts. From specific lesions in the primate hippocampus Rolls (1996) recorded responses to cued behavior at limited sites. He then applied the computational constraints back onto the hippocampus to begin to explain the role of the hippocampus in episodic memories. Elements of episodic memory include encoding of relative spatial position, temporal sequencing, novelty, and the representation of objects and faces (Derdikman and Knierim, 2014), but how the hippocampal subregions of the dentate gyrus (DG), CA3, and CA1 accomplish these encoding-decoding functions remains poorly understood. Here, we improve access to activity of single neurons and their axons in small cultured networks of DG connected to CA3 subregions in 2D over an electrode array.

In general, information transmission between two neurons has focused on mechanisms based on either a temporal code of precise timing or more commonly a rate code of frequency of the action potentials (Pimashkin et al., 2016). Here, we extend findings on rate codes in individual regions to the intermediate scale of small populations and their network interactions, especially in the specific hippocampal sub-regions of dentate gyrus (DG) and CA3, focusing on decoding processes at network levels. In particular, since Wixted et al. (2014) considered sparse distributed coding as the most efficient way for hippocampal neurons to rapidly encode episodic memories, we classified the spike rates extracted from the neural assemblies as highly or sparsely distributed.

From a methodological point of view, we first separately dissociated hippocampal cells from DG and CA3 sub-regions. Then, we co-cultured the neurons within a two-chamber device on a Multi-Electrode Array (MEA) with axonal connectivity via micro-tunnels (Brewer et al., 2013). The rationale was to reproduce a reduced model for a hippocampal DG-CA3 circuit that provided broad accessibility, useful to fill some of the gaps typical of *in vivo* models: this experimental set-up, for example, provided a more specific platform for continuous access to development, self-organization, stimulation, and measurements at multiple sites. In particular, these cultured networks permitted continuous access to 44 stimulation and 60 recording sites, increased control of structural connectivity, and network manipulation to complement *in vivo* models (Brewer et al., 2013; Poli et al., 2015). Finally, we tested the hypotheses that, in our engineered networks, DG neurons preferentially communicate information to CA3 and that the information sources in DG can be decoded by the evoked responses in CA3 (target).

Neurophysiologists often approach these questions through multiple presentations of specific stimuli applied in different sites (i.e., sources of the information) and by measuring the stimulus-response curves for encoding function. Conversely, given the output spike train (i.e., the evoked responses in target region) the decoding function defines the stimuli (Rieke et al., 1997). Repetitions of specific stimuli allow observing the repeatability of the evoked responses and, therefore, the capability of the target activity to identify (i.e., to decode) the information source. For this reason we applied specific electrical stimuli, repeated 25 times at each of 22 different sites in each sub-region (Ide et al., 2010). We used a paired-pulse stimulation protocol (Bouteiller et al., 2010) to activate silent synapses, to evoke time-locked activity and to induce more efficient information transmission over the multi-synaptic network. Our co-cultured experiments showed sparse representation of information in CA3, specific for the different stimulation sites in DG. Furthermore, we found that target responses significantly decoded the information sources, especially when stimulation sites in DG evoked activity in CA3.

## Materials and methods

### *In vitro* neural networks

**Figure 1:**
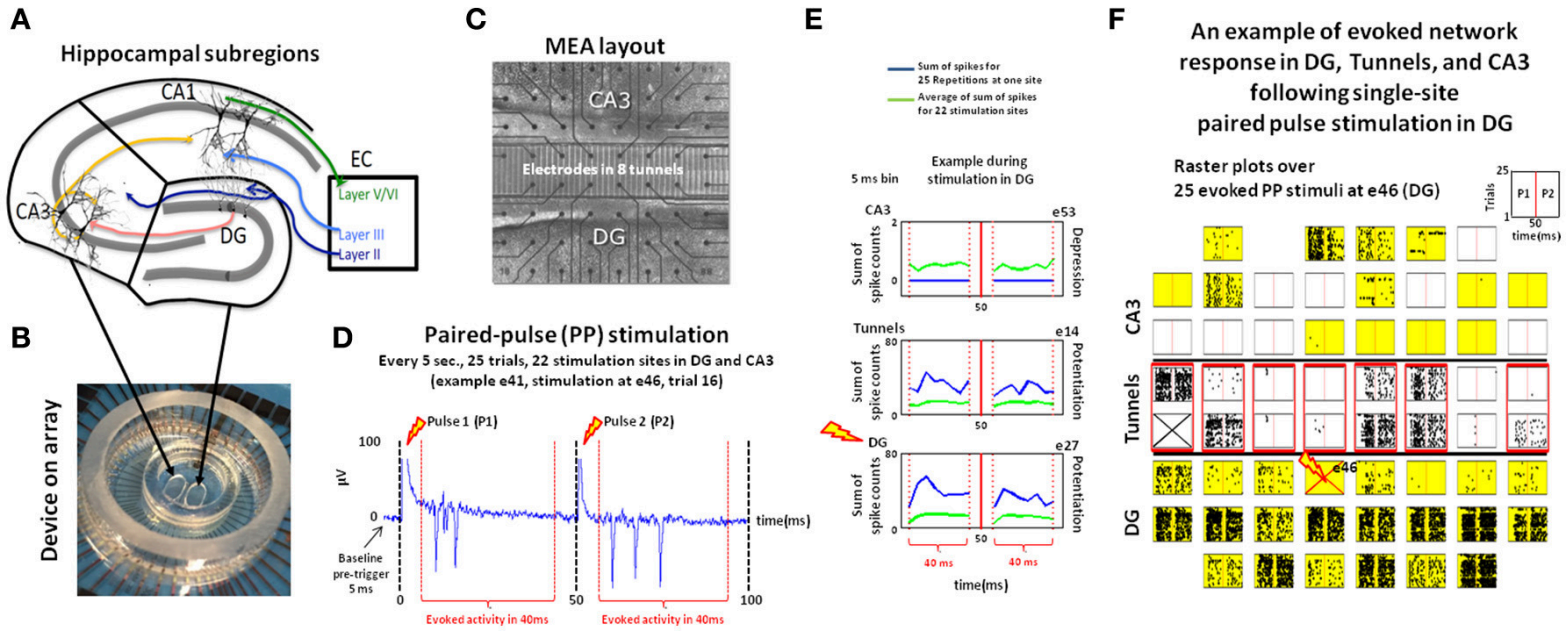
**Experimental design**. Hippocampal DG and CA3 neurons **(A)** were co-cultured and communicated through axonal connectivity via micro-tunnels using a two-chamber PDMS device **(B)** on a multi-electrode array (MEA) to measure the properties of the network response during transmission from one region to the other. **(C)** Neurons were co-cultured using two-chamber PDMS device on a MEA: 22 electrodes each for CA3 and DG with 15 electrodes in 8 tunnels **(D)** Example of extracellular recording of the burst of action potentials recorded at electrodes 41 (e41) in CA3 during paired-pulse stimulation delivered at e46 in DG. **(E)** Example post-stimulus-time histograms representing the response at electrodes e53 (CA3) and e27 (DG), and tunnel e14 evoked by paired-pulse stimulation at e46 in DG (blue lines) relative to the average response for those electrodes evoked by all stimulation sites in that region (green line). The activity after the first pulse (P1) and second (P2) pulse are separated by a vertical red line. Here, a paired-pulse stimulus in DG produces above average responses recorded at DG e27 (bottom) and tunnel e14 (middle), but a zero response at CA3 e53 (top). **(F)** Raster plot showing 100 ms of the response recorded at each electrode among the 8 × 8 electrode grid and each stimulation trial vertically stacked in each sub-panel for each electrode following paired-pulse stimulation to e46 (red cross on the bottom). Electrode 31 (top, prior stimulation) was excluded from the analysis due to amplifier saturation. Electrodes activated by at least one stimulation site in DG region are shaded in yellow for CA3 (top) and DG (bottom), red boxes for tunnels. In this co-culture, stimuli delivered at electrode 46 (bottom) sparsely activated a subset (6) of the total number of active electrodes in CA3, sites that were also specific for that stimulus location (Array 17210).

The procedure was has been described in Brewer et al. (2013). Briefly, the hippocampus was removed intact from the overlying neocortex of each hemisphere of postnatal day 3 rats before dissection of the sub-regions. First CA1 was separated from DG-CA3 by making cuts along the DG-CA1 boundary using DG rostral and ventral ends as anchors. Then CA3 was separated from DG using clearly visible boundaries at 30x under the dissecting microscope (Olympus CKX41) in a laminar flow hood (Figure 1A). To create a dual-compartment configuration, a poly-dimethyl-siloxane (PDMS) device was aligned over the 60 planar TiN/SiN microelectrodes of an MEA60 (30 μm diameter, 200 μm spacing; MultiChannel Systems, MCS, Reutlingen, Germany) (Figure 1B). The chambers were interconnected by 51 micro-tunnels with dimensions 3 × 10 × 400 μm, allowing axonal growth but not migration of cell bodies or traversal by dendrites (Claverol-Tinture et al., 2005; Pan et al., 2014). Tissues from each hippocampal subregion were subjected to a brief digestion in papain, followed by trituration into a suspension of single cells. The primary DG neurons were plated at a density of 1000 cells/mm^2^ in one well, followed within minutes by plating primary CA3 cells into the other well at 330 cells/mm^2^ to mimic the *in vivo* anatomical density ratio of 3:1 for DG-CA3 (Braitenberg, 1981; Figure 1C). Intrinsic neuronal properties allowed the native directionality of DG to CA3 not only when DG cells were plated and incubated before plating the target cells (CA3; Pan et al., 2011) but also, as here, with nearly simultaneous plating (Bhattacharya et al., 2016). Neurons were maintained in culture for up to 5 weeks in NbActiv4TM medium (Brewer et al., 2008; Bhattacharya et al., 2016; BrainBits, Springfield, IL, USA) in a humidified incubator having an atmosphere of 5% CO_2_ and 9% O_2_ at 37°C. DG-DG and CA3-CA3 networks were used as controls. During the recording sessions the cultures were placed in the MEA60 amplifier at constant temperature (37°C) and in a humidified atmosphere of 5% CO_2_ and 9% O_2_ (custom gas mixture, balance N_2_; Airgas, Santa Ana, CA) to avoid evaporation and pH drift. The recordings were made in 5 engineered networks during the third week of culture at 25 kHz sampling frequency with 1,100x amplification and a hardware filter of 1–3,000 Hz. At the time of recording, we counted live neurons from photos using ImageJ to determine the number of surviving cells under our culture conditions.

### Spike detection

In this work we used the peak-to-peak algorithm described in Maccione et al. (2009). A differential threshold, equal to 8 times the standard deviation of the baseline noise, was applied independently for each channel. The peak lifetime and the refractory period were set at 2 and 1 ms, respectively. Spike sorting did not significantly increase the spatial reconstruction of the network since we were sampling the activity of a few thousand neurons with only 60 microelectrodes. For this reason the raw data were not spike sorted.

### Stimulation protocol

Paired-pulse stimulation (Figure 1D), classically known to facilitate pre-synaptic transmitter release and increase the efficacy of stimulation (Bouteiller et al., 2010), promotes information transmission (Ide et al., 2010). In this paper the stimulation trains included two groups of 30 μA constant current paired pulses (biphasic, 100 μs/phase duration beginning positive, 50 ms between individual stimuli). To minimize the chance of nearby facilitation, the first group was applied to one specific well site on the top, the second one on the bottom. This alternation was repeated 25 times for all electrodes in both chambers. A wait period of 5 s was inserted between the pairs to minimize plasticity effects of the prior stimulation, whose site was excluded from our analysis due to amplifier saturation. Stimulation artifacts were removed by subtracting a best-fit exponential from each stimulus. In order to avoid stimulation artifacts, we chose a 5 ms blanking window after each pulse and evaluated the evoked spike rates defined as the number of spikes recorded by each electrode during the following 40 ms over 25 repetitions divided by the total time (i.e., 2 s for 80 ms × 25 repetitions). The rationale of this choice was due not only to the latency of spikes that increased from 2 (i.e., the earliest responding spike that might occur) to 10 ms with increasing distance from the stimulus, but also because the amplitudes of action potentials that decreased to near baseline after 40–45 ms from that stimulus (Ide et al., 2010). This blanking period of 2–5 ms after stimulation is commonly used in MEA work to compensate for the distortion of the signal from the electrical stimulus's effect on the amplifier (Wagenaar and Potter, 2002; Wagenaar et al., 2004). Finally, out of the two electrodes within each tunnel, we chose the one with higher spike rate, assuming it to have better axon to electrode coupling and hence a more accurate reporter of axon activity.

We considered whether the activity on each active electrode (spike rates > 1.5 Hz) was due to the application of the stimulus and not to the spontaneous baseline activity (Chiappalone et al., 2008). We assessed baseline activity by two methods. The first was the activity in a 3 min. recording immediately prior to the application of the paired pulse stimulation protocol. The second way we measured baseline rates was to count the number of spikes recorded by each active electrode in the 5 ms before paired-pulse stimuli delivered at 22 different sites in both chambers for 25 times (one trial is shown in Figure 1D).

### Kurtosis measure

A sparse code is one in which relatively few coding units (electrodes) are active at any one time to code any specific stimulus (Kanerva, 1988; Willomore et al., 2000). To quantify the sparseness of the evoked responses we used the kurtosis of the spike rates, which is the most commonly used selectivity index (Willmore and Tolhurst, 2001; Lecky et al., 2005). This measure is defined as the normalized fourth moment of the spike-rate distribution, subtracting three in order to have a kurtosis of zero for the Gaussian case:

(1) $$K=\;\frac{\sum_{i\;=\;1}^{N_{e}}\left\{\frac{1}{N_{stim}}\sum_{j\;=\;1}^{N_{stim}}{\left(\frac{SR_{i,j}-\mu_{i}}{\sigma_{i}}\right)}^{4}\right\}-3}{N_{e}}$$

where *N*_*e*_ is the number of electrodes in one target well (i.e., 22 for each chamber), *N*_*stim*_ is the number of the stimulation sites (also, 22 for each chamber), *SR*_*i, j*_ is the spike rate recorded by the *i*^*th*^ target electrode for the *j*^*th*^ stimulation site, μ_*i*_ and σ_*i*_ are the mean and standard deviation of *SR* over all stimulation sites for the *i*^*th*^ target electrode, respectively.

### Uniqueness curves for specificity of target responses

A good code needs to approach uniqueness of target responses to specify different stimuli. In order to study this specificity of the neural code, we first considered the target electrodes with the highest spike rate evoked by each stimulation site. If one of these electrodes in CA3 was the most active for a single stimulation site but not for the other ones, its response was considered 100% unique. If this electrode was the most active for two stimulation sites and no others, then its uniqueness was ~95% [1−(2−1)/(22−1); see Equation 3]. Then, in an incremental approach, we iteratively considered the target electrodes with the 2, 3, 4, …up to 22 highest spike rates evoked by each stimulation source. Finally, for each group size (i.e., 1, 2, 3, 4, …up to 22) of these most active electrodes, we averaged the percentages over all 22 target electrodes to generate an average regional uniqueness. The rationale of this analysis was to create a uniqueness curve that allowed evaluating uniqueness vs. the number of the highest spike rates involved among the target electrodes.

**Figure 2:**
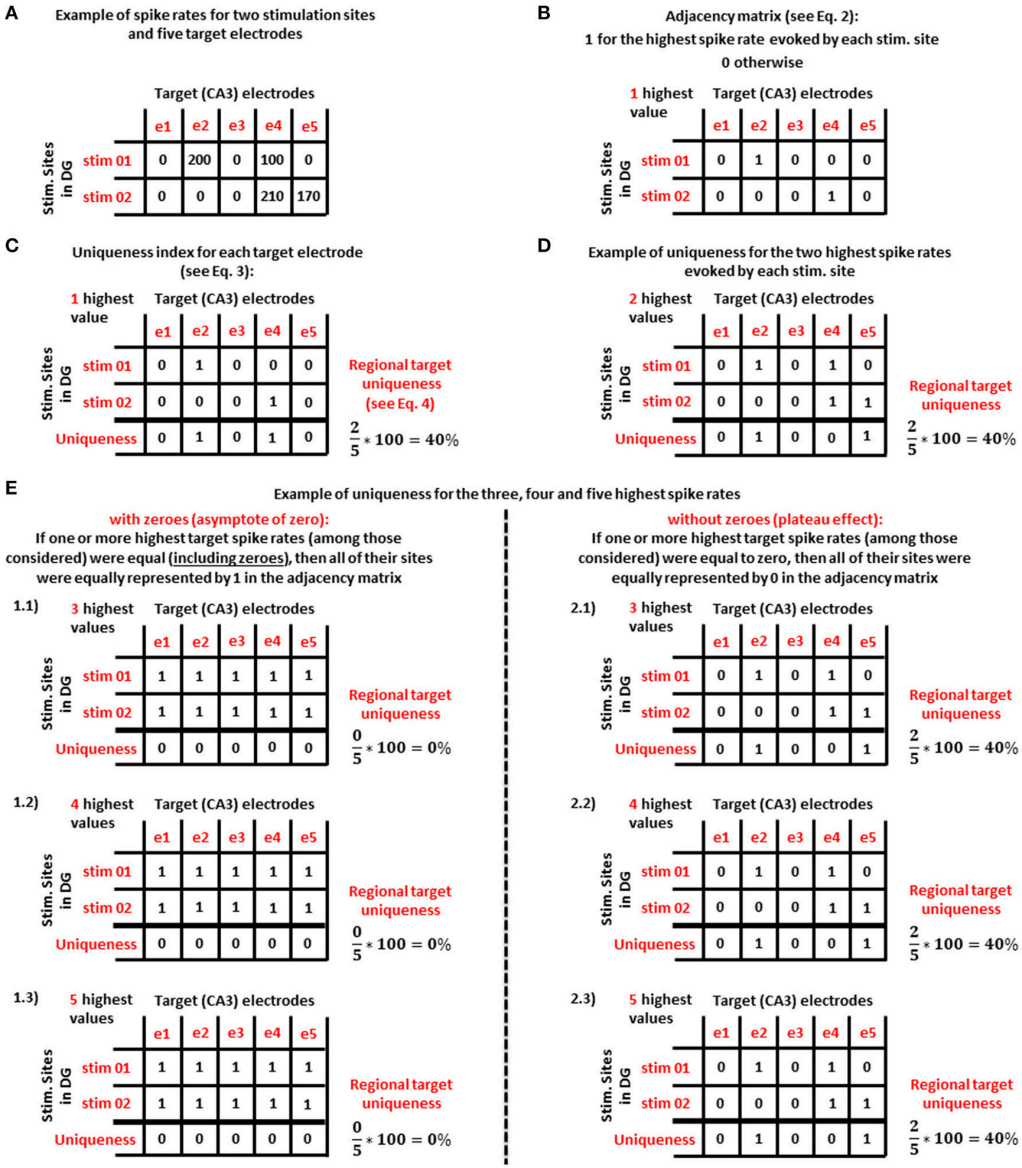
**Example of the uniqueness analysis method for the most active electrodes in the target (CA3) region that are specific to one stimulation source in DG. (A)** Uniqueness analysis applied to the spike rates (in Hz) of five target (CA3) electrodes for two stimulation sites in DG. **(B)** Adjacency matrix based on the highest spike rate evoked by each stimulation site shown in panel **(A)**. **(C)** For panel **(B)** adjacency matrix, uniqueness index for the *i*^*th*^ target electrode and regional target uniqueness (i.e., the average of the uniqueness measures over all target electrodes). **(D)** Considering the two highest spike rates evoked by each stimulation source, a new adjacency matrix yields the same regional average in this example. **(E)** Uniqueness analysis considering the target electrodes with the 3 (**E** 1.1 and 2.1), 4 (**E** 1.2 and 2.2), and 5 (**E** 1.3 and 2.3) highest spike rates evoked by each stimulation source. By considering zeroes (1.1, 1.2, and 1.3) uniqueness reaches an asymptote of zero. By excluding zeroes (2.1, 2.2, and 2.3), uniqueness produces a plateau.

Figure 2 shows an example of this approach for only two stimulation sites and the spike rates for five target electrodes (Figure 2A). Formally, we let *A*_*j, i*_ to be an adjacency matrix (Equation 2) with *j* stimulation sites in DG and *i* target (CA3) electrodes:

(2) $$A_{j,i}=\{\begin{array}{cc} 1 & \;\;\;for\;the\;highest\;spike\;rate\;evoked\;by\;each\;stim\;\;site\\ 0 & otherwise \end{array}$$

From this adjacency matrix (example in Figure 2B) we first extracted 22 uniqueness indices (*U*_*i*_, Equation 3) for each target electrode:

(3) $$U_{i}=\{\begin{array}{c} \;\;\;\;\;\;\;\;\;\;\;\;\;\;\;0\;\;\;\;\;\;\;\;\;\;\;,\;\;\;\;\;\;\;\sum_{j\;=\;1}^{N_{stim}}A_{j,i}=0\\ \left[1-\left(\frac{\left(\sum_{j\;=\;1}^{N_{stim}}A_{j,i}\right)-1}{\left(N_{stim}-1\right)}\right)\right],\;\sum_{j\;=\;1}^{N_{stim}}A_{j,i}\neq 0 \end{array}$$

where *N*_*stim*_ was the number of the stimulation sites.

Then, we averaged these indices into a regional uniqueness measure (*%U*) defined as follows:

(4) $$\%U=\frac{\sum_{i\;=\;1}^{N_{e}}U_{i}}{N_{e}}*100$$

where *N*_*e*_ was the number of target electrodes. Examples of the uniqueness indices and regional target uniqueness begin in Figure 2C.

Finally, by increasing the number (*n*) of the highest spike rates of the target electrodes evoked by the *jth* stimulation source, we obtained *n* different adjacency matrices: one adjacency matrix for 1 highest spike rate (Figure 2B), one for the target electrodes with the 2 highest spike rates (Figure 2D) and so on (*n*_*max*_ = 22, as many as the electrodes in each well). From each of these adjacency matrices we extracted the uniqueness indices and the regional target uniqueness in order to create the uniqueness curve. This curve was evaluated in two ways. (1) We considered zero as an informative value, which is especially important when an electrode was active for some stimulation sites but not others (example starting in Figure 2E1.1). If one or more highest target spike rates were equal (including zeroes), then all of their sites were equally represented by 1 in the adjacency matrix. (2) In a second approach, we discarded zero activity responses in order to consider only active target electrodes (example starting in Figure 2E2.1). In this case, if one or more highest target spike rates were equal to zero, then all of their sites were equally represented by 0 in the adjacency matrix.

By observing how the uniqueness indices (i.e., y axis of the curve) changed by increasing the number of highest spike rates in the target region (i.e., x axis of the curve), we detected the maximum percentage of specificity (i.e., peak of the curve). In terms of rate code, this peak quantified the number of target (CA3) electrodes needed to optimally code for each stimulation site in DG (see Section Uniqueness of CA3 Responses to Stimulation at Specific Sites in DG, **Figure 7**).

### Decoding of stimulation site during classification of evoked activity using support vector machine learning (SVM)

While the uniqueness approach (Section Uniqueness Curves for Specificity of Target Responses) quantified the specificity of the target responses for each stimulation source, it provided little information about the degree to which activity in each evoked response could be used to determine (i.e., decode) the identity of the stimulation site. To quantify the likelihood that a certain set of target (CA3) electrodes decoded the source of the stimulus, we used learning algorithms for classification (support vector machine, SVM) in Python code (Python scikit_learn library v0.17-5, default parameters for a linear kernel). SVM was trained from a stratified sample of 80% of the trials and decoding performance determined by testing (i.e., classifying) the remaining 20% of trials. Our cross-validation training-testing methodology included training sets based on selections of 2, 5, 10, and 15 electrodes at time. For example, if two of the 22 electrodes are chosen at a time for analysis there are then 20 remaining electrodes available for classification. Each potential two electrode combination must then be classified and average classification performance computed. The permutations of 22 channels in all combinations of five electrodes at a time resulted in 26,334 combinations that must be computed. In order to reduce the computational time required for conducting the full channel combination set with 10 (646,646) and 15 (170,554) channels, we limited the number of possible combinations to 26,334, randomly drawn from the pool of all possible pairs.

## Results

By analysis of spike rates in response to paired-pulse stimuli, we addressed a number of questions about coding and decoding from a DG to a CA3 neural population (and vice-versa). What is the CA3 neural code for each stimulation site in the DG region (i.e., the neural coding in the target region relative to the source)? Is the neural code in our CA3 co-cultures “sparse”? How do the target (CA3) neurons decode the sources? In order to answer these questions we studied the information transmission between the DG and CA3 regions by means of a two-chamber device with axonal connectivity via micro-tunnels. DG-DG and CA3-CA3 networks were used as controls. In our 5 engineered DG-CA3 co-cultures, we determined cell survival of 375 ± 50 cells/mm^2^ in CA3 and 1075 ± 90 cells/mm^2^ in DG, values that were consistent with excellent neuron survival and equivalent to the original cell plating densities of 330 and 1,000 cells/mm^2^, respectively. Our engineered networks maintained 84% axonal polarity of DG to CA3 (Bhattacharya et al., 2016; consistent with the hippocampal anatomy). In contrast, the control networks did not show a preferred direction of spike propagation. In this work, we applied paired-pulse stimuli (Bouteiller et al., 2010), repeated 25 times at each of 22 different sites in each sub-region (Ide et al., 2010). The rationale of this choice was to activate silent synapses, to evoke time-locked activity and to induce more efficient information transmission over the multi-synaptic network. Regional baseline rates for our co-cultures during the pre-trigger period (5 ms before each paired-pulse stimulus) over 25 stimulation trials were 11.2 ± 2.3 Hz in CA3 and 22.2 ± 2.8 Hz in DG. Similar values were obtained for 3 min of spontaneous activity: 12.2 ± 2.4 Hz in CA3 and 23.2 ± 2.1 Hz in DG. The rates during stimulated activity were 4–5 times larger at 55.7 ± 3.5 Hz in CA3 and 86.5 ± 2.6 Hz in DG, over all active electrodes in each chamber. These large rate differences with baseline 22% of the evoked rate are similar to the 20% spontaneous threshold used by Chiappalone et al. (2008) to discriminate the evoked responses from the spontaneous fluctuations of cortical activity level. We include this information for historical comparisons, but submit two reasons that may decrease their relevance. First, the distribution of inter-spike intervals (ISI = 1/spike rate) fits a power law (log ISI vs. log prevalence is linear; Bhattacharya et al., 2016), so an average may not accurately summarize the distribution. Second, since the stimulation protocol can activate inhibitory as well as excitatory neurons, a single-sided evaluation above baseline would exclude some stimulation sites that evoke less spikes (Figure 1E, blue line below the green line average for other sources). The spiking activity of one of these co-cultured networks is shown as an example in Figure 1F (raster plots over 25 evoked paired-pulse stimuli at electrode 46). Electrodes activated by at least one stimulation site in DG region are shaded in yellow for CA3 (top) and DG (bottom), red boxes for tunnels. In this co-culture, stimuli delivered at electrode 46 (bottom) sparsely activated a subset (~6 electrodes) of the total number of active electrodes in CA3, sites that were also specific for that stimulus location. Generally, the evoked responses in these engineered networks followed three types of patterns with repetition: (1) similar responses with each trial (Figure 1F), or (2) responses in early trials that were extinguished for later trials, or (3) the reverse. The source of these variations will be not studied in this paper.

### Spike rate analysis

**Figure 3:**
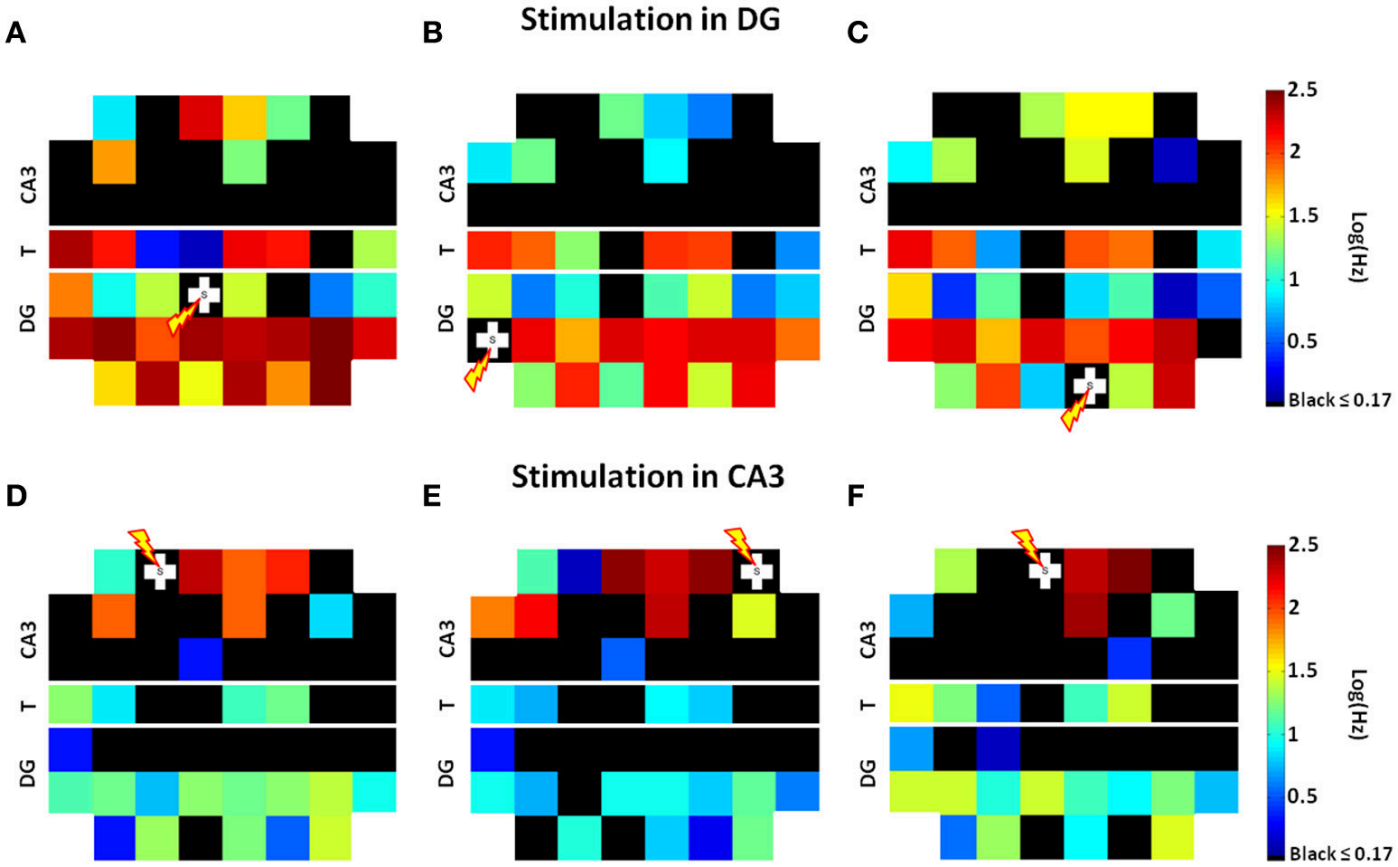
**Stimulation at one site in DG activates a large set of DG neurons with high axonal transmission through the tunnels (T) to activate a specific smaller set of electrodes in CA3**. Spatial distributions of average spike rates at each electrode on a standard MEA60 layout from stimulation at three specific sites in DG [white crosses **(A–C)**] show that specific small sets of electrodes in CA3 are active, consistent with sparse coding. Stimulation in CA3 is less effective in activating tunnel axons and less effective in driving these axons to activate DG than when DG was stimulated **(D–F)**. In each panel, from the two electrodes in the 8 monitored tunnels (see Figure 1C), we choose one with higher spike rate due to better axon to electrode coupling (Array 17210).

Since Pimashkin et al. (2016) found that spike rate measures could be used to retrieve information not only about the neural code but also about the stimulus location, we analyzed the spike rates evoked by paired-pulse stimulation during 25 trials at each electrode of both chambers. Because the restricted 3 μm height of the microtunnels promoted axon growth but did not allow in-growth by neuronal somata, the recorded tunnel activity reflected the information transmission by axons between source and target (Pan et al., 2014). Figure 3 shows the spatial distributions of these spike rates on a standard MEA60 layout from three stimulation sites in DG (Figures 3A–C). Here we found widespread activation in the DG subregion, frequent activation of axons in the tunnels and most importantly, activation of a small, specific set of electrodes in CA3, consistent with sparse coding. Stimulation in CA3 (three examples of stimulation at CA3 sites are shown in Figures 3D–F) was less effective in activating the CA3 subregion and tunnel axons, and in driving these axons to evoke responses in DG than when DG was stimulated.

**Figure 4:**
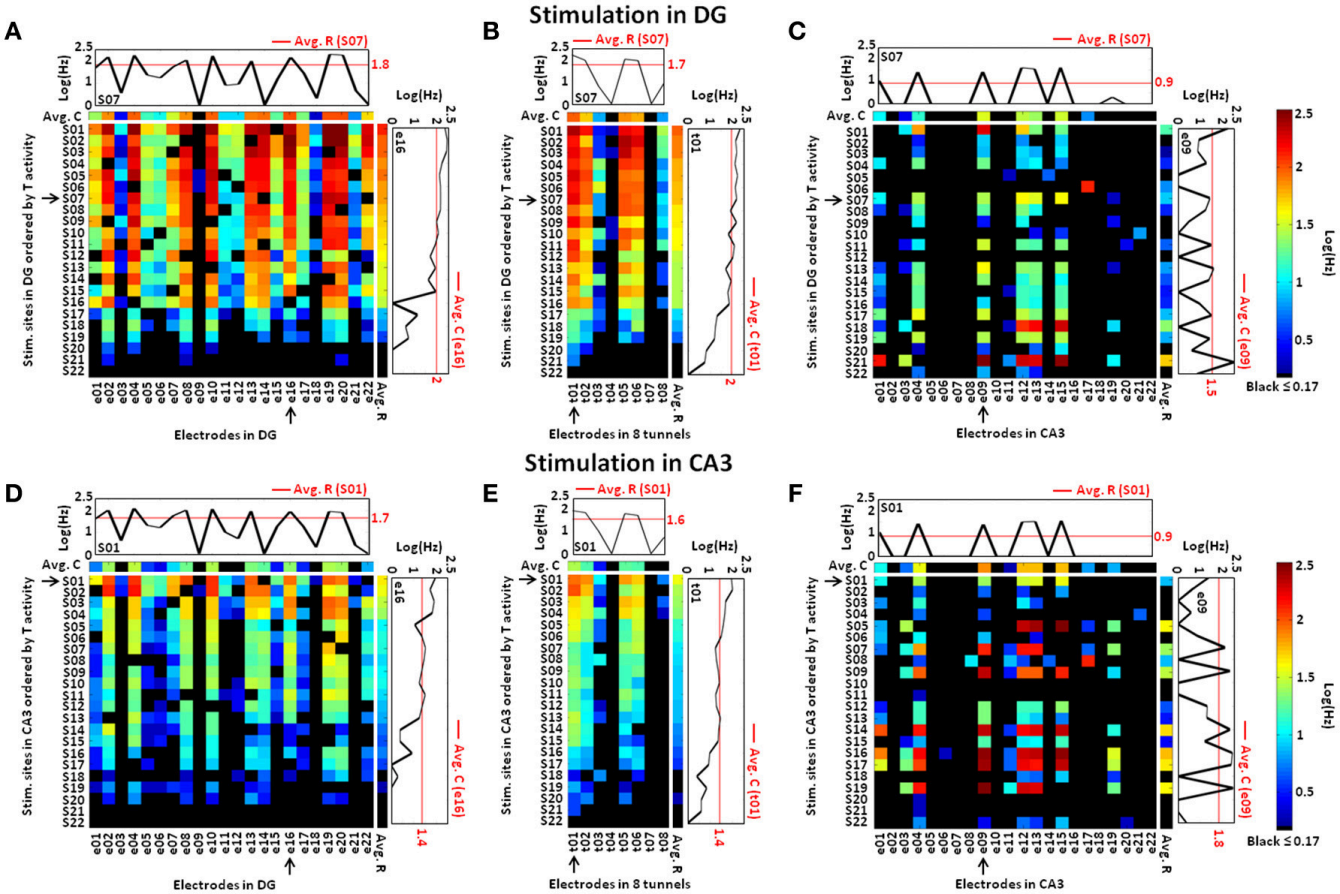
**Stimulation of each site in DG or CA3 activates a more limited set of electrodes sites in CA3 than occurred in DG, suggesting sparse coding of the neural information. (A)** Specific stimulation of DG at each site (row) evokes responses in DG transmitted through the tunnels **(B)** that activate a relatively small set of responses in CA3 **(C)**, suggesting sparse coding. Compared to stimulation of DG, stimulation in CA3 evokes less activity in the DG region **(D)** and tunnel axons **(E)**, despite higher activity in CA3 **(F)**. Therefore, CA3 stimulation is less effective in driving the axons in the tunnels to activate DG than when DG is stimulated. The rows of each spike rate matrix are ordered according to the spike rates in the tunnels (T) from highest (top) to the lowest (bottom). The log of the averages of the spike rates across recording sites for each stimulus location (i.e., the averages of each row, Avg. R) is shown to the right of the vertical white line in each spike rate matrix. Similarly, the averages across stimulation sites (i.e., the averages of each column, Avg. C) are shown and color coded on a log scale above the horizontal white line. Responses to two particular stimulating electrodes, highlighted by an arrow (S07 in DG, S01 in CA3), are compared to the average for that target electrode. This shows evoked responses either greater or smaller than the average at each recording electrode. Responses at three recordings sites (see arrows at e16 in DG, t01 in the tunnels and e09 in CA3) also show that a single recording electrode reports both upward and downward modulation of spike rates, relative to the average, as the stimulus location changes. This is also consistent with sparse coding in CA3 **(C,F)** (Array 17210).

To represent all stimulation sites, we assembled the responses evoked by 22 paired-pulse stimuli during 25 trials into spike rate matrices (Figure 4) in which rows were the stimulation sites and columns were the electrodes in each compartment, source, tunnel or target (Jimbo et al., 1999; Pimashkin et al., 2016). To assist in visual evaluation, the row order of the stimulus locations was determined by the activity evoked in the tunnels (high to low), as seen for DG (Figures 4A–C) and for CA3 (Figures 4D–F). During stimulation in DG (Figure 4A), as in spontaneous activity (Bhattacharya et al., 2016), the axon activity in the tunnels (Figure 4B) was dominated by the source (DG) activity showing strong information transmission through the tunnels to the target (CA3; Figure 4C). While stimulation of specific sites in DG activated a large set of electrodes in DG, a relatively small set of electrodes were activated in the target (CA3), suggesting a sparse representation of information. During stimulation in CA3 (Figure 4F) DG activity (Figure 4D) was diminished due to lower transmission through the tunnels (Figure 4E) than when DG was stimulated. For comparison, the log of the averages of the spike rates across recording sites for each stimulus location (i.e., the averages of each row, Avg. R) is shown to the right of the vertical white line in each spike rate matrix, while the averages across stimulation sites (i.e., the averages of each column, Avg. C) are shown in log scale above the horizontal white line. Further, one example of both averages is plotted graphically on a log scale both to the right and above each main graph (red lines), enabling comparison with the rates for one recording or one specific stimulation site (black lines). As suggested by Jimbo et al. (1999), the rationale of this analysis was to indicate different pathways of activity by observing the variations of the evoked responses compared to both averages. Our results showed a mixture of increased and decreased activity in each subregion, indicating different excitatory or inhibitory pathways to specific targets for a given stimulus (e.g., site S07 in DG, site S01 in CA3). Furthermore, the profile of the responses evoked by all stimulation sources in each site showed, especially in CA3, more decreased than increased activity, consistent with sparse coding (e.g., electrode 16 in DG, electrode 9 in CA3 and tunnel 1).

**Figure 5:**
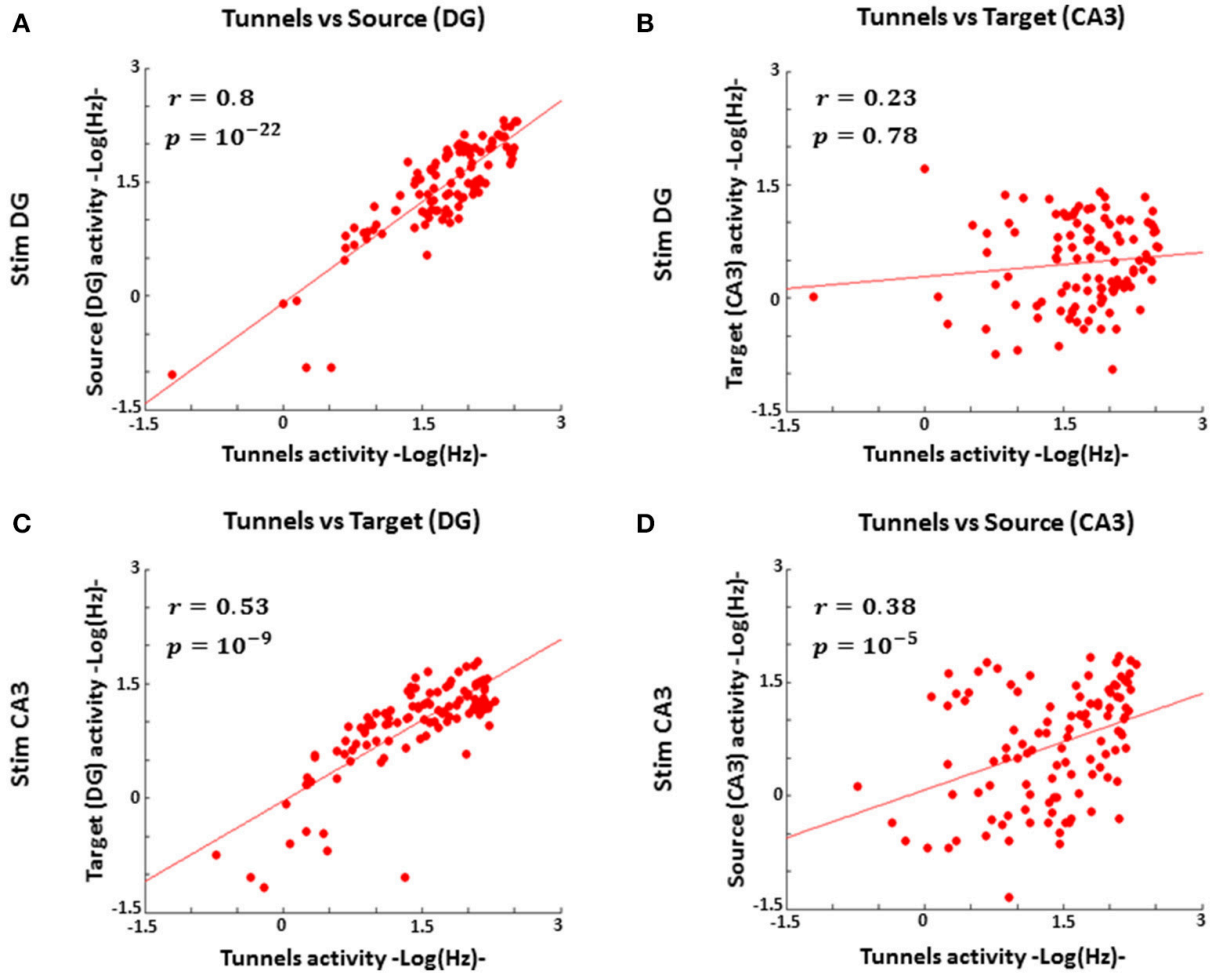
**DG spike rates correlate with tunnel activity during stimulation in DG, but the tunnel input activity to CA3 correlates poorly with CA3 activity, consistent with mass rate coding for DG to tunnels but sparse coding from tunnels to CA3 (*n* = 5 arrays). (A)** Log activity from stimulation in DG strongly correlates with log axon transmission through tunnels (coefficient of correlation *r* = 0.8, Pearson correlation *p* = 10^−22^) **(B)** During stimulation in DG log activity in CA3 is poorly correlated with log axon transmission through the tunnels (*r* = 0.23, *p* = 0.78). **(C)** Log activity in DG, during stimulation in CA3, is proportional to log axon activity through the tunnels (*r* = 0.53, *p* = 10^−9^), once again indicating a mass action effect. **(D)** Log activity in CA3 as the source is less effective at driving activity in the tunnels (*r* = 0.38, *p* = 10^−5^).

Since our engineered DG-CA3 networks maintain 84% axonal polarity of DG to CA3 (Bhattacharya et al., 2016; consistent with the hippocampal anatomy), it was reasonable to expect a strong correlation between DG and tunnel spike rates, especially during stimulation in DG. The activity in CA3 might also correlate with tunnel activity but employ a non-sparse code for stimulation sites in DG, or be uncorrelated in a largely sparse code. A sparse code would also be expected for complex information processing in CA3 to control the output activity and prevent runaway feed-forward excitation fed by projection from CA3 to other brain circuits. The evidence in Figure 5A in which DG source activity strongly correlated with axon activity (*n* = 5 arrays) over a 1,000-fold range of spike rates (coefficient of correlation *r* = 0.8), supported information transmission from within DG to axonal output. Neural activity in the target (CA3) region, when DG was stimulated, was not correlated with tunnel activity (Figure 5B; *r* = 0.23), as expected for sparsely coded target responses. Conversely, stimulation in CA3 also evoked a proportional response between DG and tunnels (*r* = 0.53, Figure 5C), once again indicating a mass action effect, but a weaker correlation between tunnel axon activity and CA3 as the source of stimulation (*r* = 0.38, Figure 5D). The activity in CA3 as a source (i.e., CA3 during stimulation in CA3) was less effective than DG at driving the activity in the tunnels, suggesting a weaker information transmission through the tunnels than the reverse direction.

### Kurtosis measure for sparse representation of activity

**Figure 6:**
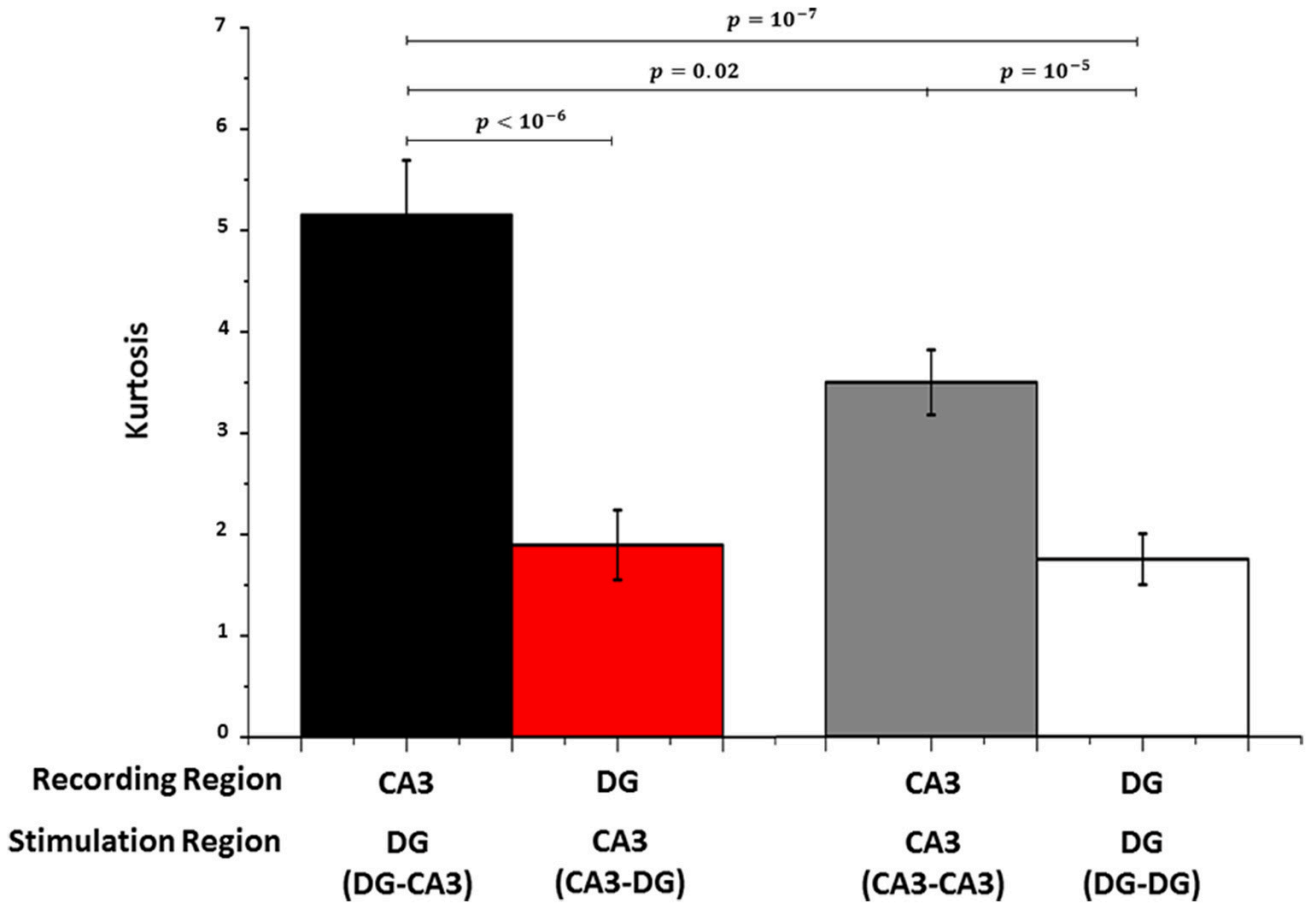
**As a measure of sparseness of spike rate coding, Kurtosis is much larger in CA3 during stimulation in DG (black bar) than DG when CA3 is stimulated (red bar) and the controls (gray bar for CA3 in CA3-CA3 and white bar for DG in DG-DG networks)**. Wilcoxon rank sum test for *n* = 5 arrays each.

We used the kurtosis measure as a sparseness indicator of the deviation of evoked spike rates from a mean Gaussian distribution (average spike rate; see Section Kurtosis Measure, Equation 1). In 5 DG-CA3 networks (Figure 6), population neural activity in CA3 evoked by site-specific DG stimulation (CA3|DG-CA3, black bar) was more sparse (i.e., higher kurtosis index) than DG cultures when CA3 was stimulated (DG|CA3-DG, red bar). Furthermore, kurtosis measures extracted from these CA3 evoked responses (black bar) were greater than those obtained from CA3|CA3-CA3 control (gray bar). Since CA3 neurons were plated in the homologous case at the same cell density in both chambers, these results suggested that the distributed excitation of more activated DG cells was needed for a more sparse response in the target (CA3) region than the CA3|CA3-CA3 control. No statistical difference was found between DG|CA3-DG (red bar) and DG|DG-DG control (white bar). Finally, CA3|CA3-CA3 control (gray bar) showed sparseness significantly larger than DG when CA3 was stimulated (red bar). In conclusion, our results indicated that the sparseness was specific to the CA3 region, especially when CA3 was driven by DG.

### Specificity of the neural code

Is the neural code in CA3 co-cultures specific to the stimulation site in DG region? Which target electrodes activated by one stimulation site are needed to optimally specify that source? What is the decoding process shown by our cultured CA3 neurons when the stimulation is applied in the DG region? To approach these questions we analyzed first the uniqueness of CA3 responses evoked by different stimuli in DG (and vice-versa) and second, we applied a SVM to target evoked activity to decode the probability of correct identification of stimulation sources.

#### Uniqueness of CA3 responses to stimulation at specific sites in DG

**Figure 7:**
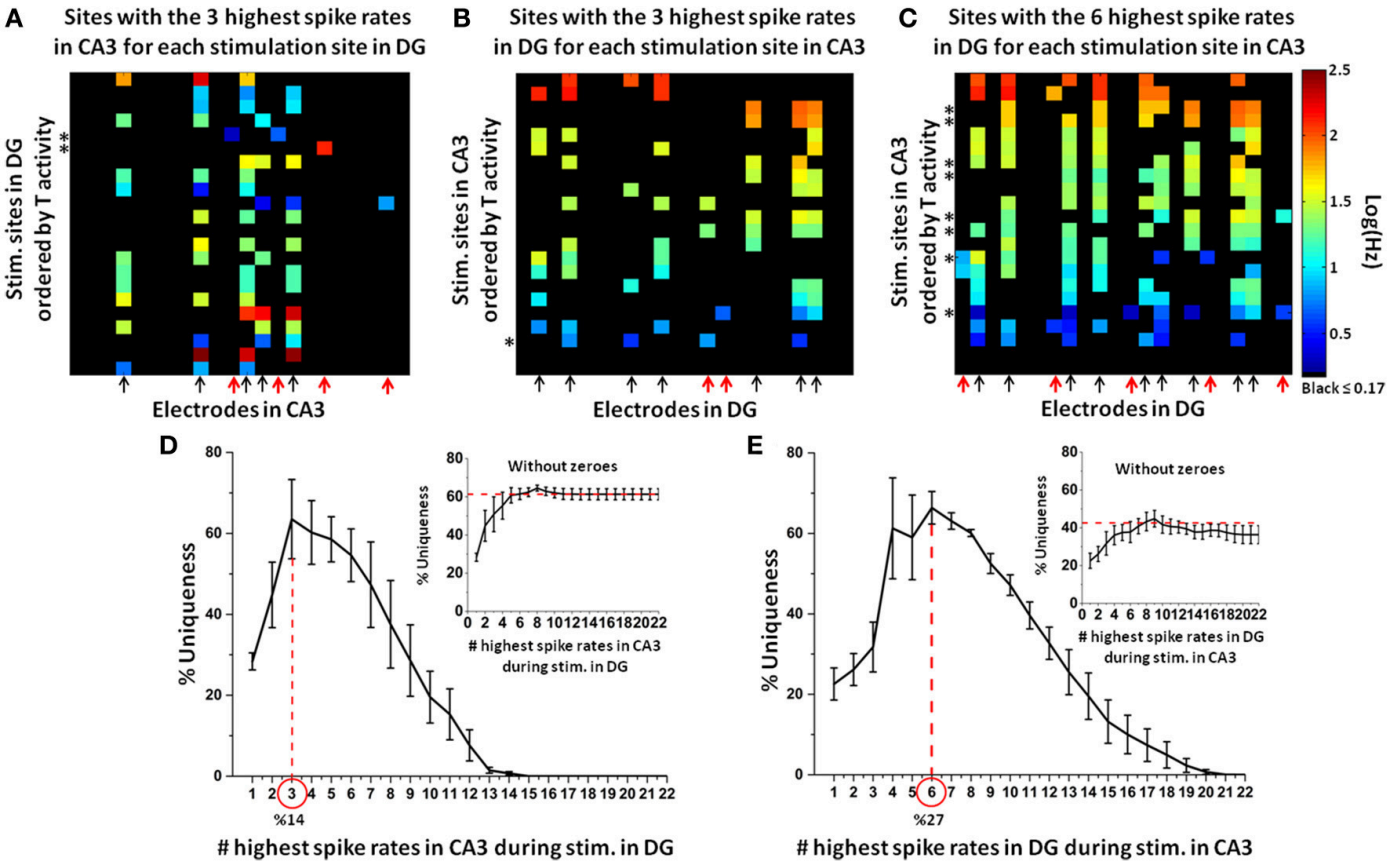
**Electrodes with the highest spike rates needed to optimally specify a unique target response to each stimulation site. (A)** Example of target (CA3) electrodes with the three highest spike rates during DG (source) stimulation (arrows mark the active sites in CA3 for all stimulation sources in DG; the red arrows indicate the four uniqueness cases >90%). Note example in which one and two responding electrodes are selected when only one or two spike rates among those considered (i.e., the three highest spike rates) are different from zero (asterisks mark the stimulation sites that evoke these responses). **(B)** Conversely, for the same network, uniqueness is lower for the target (DG) electrodes with the three highest spike rates during CA3 (source) stimulation than CA3 when DG is stimulated (arrows mark the active sites in DG for all stimulation sources in CA3; the red arrows indicated two uniqueness cases >90%). Note example in which four responding electrodes are selected when two spike rates among those considered (i.e., the three highest spike rates) are equal (asterisk marks the stimulation site that evokes these four responses) **(C)** The target (DG) electrodes with the six highest spike rates during CA3 stimulation show a higher number of unique responses than the same network presented in panel **(B)** (the red arrows indicate the five uniqueness cases >90%). Note example in which seven or eight responding electrodes are selected when two spike rates among those considered (i.e., the six highest spike rates) are equal (asterisk marks the stimulation sites that evoke these responses) [Array 17210 for panels **(A–C)**]. **(D)** For *n* = 5 arrays, electrodes in CA3 with an optimum of three highest spike rates (approximately the three most active target electrodes for each stimulation source, 3/22 = 14%) specifies 64% uniqueness of target responses to each specific stimulation site in DG (inset for the case without zeroes). **(E)** In the DG region, a similar level of uniqueness (i.e., 66%) is optimum for consideration of the six highest spike rates (inset shows the case without zeroes).

In an incremental approach, we first created an adjacency matrix of the target (CA3) electrodes with the highest spike rate evoked by each DG stimulation site. Then we iteratively increased to the recording sites with the 2 highest spike rates for each stimulation site, then 3, up to the maximum of 22 (i.e., the maximum number of target electrodes). Finally, from these adjacency matrices, we derived a uniqueness curve for each network configuration (approach illustrated in Figure 2). By using the target electrodes with the three highest spike rates in CA3 (Figure 7A) and DG (Figure 7B) for each stimulation source in either DG or CA3, respectively, we found nine active sites in each target region over all stimulation sites (red arrows mark four uniqueness cases >90% in CA3, two in DG). The evoked responses to the stimuli were much more specific in CA3 than DG. Conversely, the uniqueness of the evoked activity in DG during the stimulation in CA3 was optimal for the electrodes with the six highest spike rates for each stimulus (arrows indicated fourteen active sites in DG over all stimulation sites in CA3; the red arrows mark five cases with uniqueness >90%; Figure 7C). The finding of three and six highest spike rates in these figures was based on the peak of the uniqueness curves obtained from 5 arrays (Figures 7D,E). The optimum uniqueness of 64% used only the target (CA3) electrodes with the three highest spike rates (approximately the three most active target electrodes for each stimulation source; 3/22 = 14%) to specify the stimulation site in DG (Figure 7D, main plot). In the DG region, maximum uniqueness of 66% was reached using the recording electrodes with the six highest spike rates (approximately the six most active target electrodes for each stimulation source; 6/22 = 27%) evoked by the stimulation sites in CA3 (Figure 7E, main plot). Therefore, based on a relatively small set of target electrodes with the three or six highest spike rates, the optimally unique responses to stimulus indicated, especially in CA3, site-specific coding of the neural information transmitted from one source to the target. Finally, if we discarded zero activity responses in order to consider only active target electrodes, the plateau of uniqueness levels in CA3 during stimulation in DG (inset Figure 7D) was significantly higher (61%) than that found in DG when CA3 was stimulated (42%, inset Figure 7E). Also in this case (i.e., excluding zeroes), the higher uniqueness in CA3 than DG appears due to lower specificity in DG than CA3.

#### Support vector machine to identify the stimulation source in DG from evoked CA3 responses

**Figure 8:**
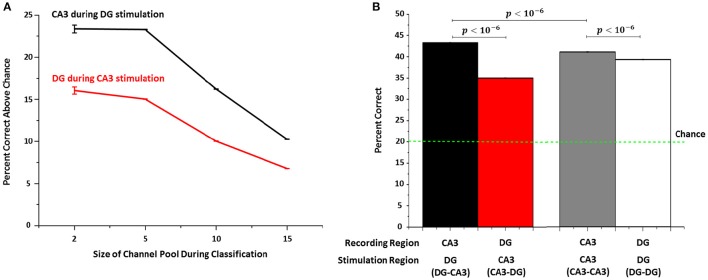
**Support vector machine (SVM) shows that target (CA3) responses permit identification of the stimulation site better than chance when the source of the stimulus is in DG. (A)** The percentage above chance varies with the number of channels to be identified (“permuted channels”) in CA3 during DG stimulation (black line), which is consistently higher (Mann-Whitney test: *p* < 10−6) than for responses measured in DG when CA3 is stimulated (red line) (*n* = 5 arrays each). **(B)** For example, when identifying the source among five sites in DG from recordings in CA3, SVM can correctly identify the stimulation site in 43% of trials when DG is stimulated and CA3 recorded compared to 34% of trials when CA3 is stimulated and DG recorded. Performance for CA3-CA3 control networks (gray bar) and DG in DG-DG networks (white bar) are intermediate.

We used SVM learning to quantify how well stimulation sites can be identified based on target responses in the opposite well for 25 trials. In order to reduce the computation time required for SVM analysis of all possible combinations, we examined how well SVM performed on subsets of the data. In the simplest subset, we determined how well the recording electrodes could be used to distinguish between a pair of stimulating sites, using all permutations of pairs of recording and stimulation sites. This was repeated for 5, then 10 and then 15 stimulation sites. For groups of 2, 5, 10, and 15 channel permutations, chance performance would be 50%, 20%, 10% and 7% correct responses. Regardless of the group size, identification of the correct stimulation site in DG from evoked CA3 responses was significantly above chance (Figure 8A, black line, 23 to 10%) and greater than the reverse of identification of stimulation sites in CA3 from evoked DG responses (red line, 16 to 7%). Focusing on the group of 5 target permutations with 20% chance, Figure 8B shows the percent correct compared to several controls. The CA3 network correctly classified (decoded) the stimulation site in DG 43% of the trials (Figure 8B, black bar; 43–20% chance = 23% in Figure 8A), which was significantly higher than decoding the reverse, i.e., identifying from DG sites the locus of stimulation in CA3 (35%, red bar). In homologous control groups, CA3-CA3 (41%, gray bar) and DG-DG (39%, white bar), identification of the stimulation site was less reliable than for the DG to CA3 connection.

## Discussion

To code and decode a portion of hippocampal function, we created subnetworks of DG and CA3 neurons connected by micro-tunnels over MEAs. Imposing these micro-tunnels (designed with dimensions that selected for axonal growth over dendrites), we obtained a model for the DG-CA3 circuit in which we previously showed that the neural populations maintained separable spike and burst dynamics, spike direction information, and biomarkers comparable to *in vivo* anatomy (Brewer et al., 2013). The structural specificity for DG-CA3 networks vs. control cases (i.e., CA3-CA3 and DG-DG) had a significant effect on connectivity: functionally, in our most recent work (Bhattacharya et al., 2016), we showed that spontaneous propagation of over 80% of the activity proceeded in the native direction from DG to CA3. These directional effects indicated self-wiring capabilities intrinsic to the neurons of the sub-regions. We also found a strong dependence of the network connectivity on number of tunnel inputs (i.e., 2, 5, 10, 15, and 51; Pan et al., 2015). Furthermore, we observed a robust connection of DG to CA3 only when a high number of tunnels was simultaneously active (Bhattacharya et al., 2016) indicating, therefore, a need for multiple tunnels (i.e., multiple axons) to evoke activity in CA3 (not a single axon or single fiber bundle). Here, paired-pulse stimulation (inter-pulse interval 50 ms) was applied at 22 different sites and repeated 25 times in each sub-region to evoke time-locked activity. DG-DG and CA3-CA3 networks were used as controls. As in spontaneous activity (Bhattacharya et al., 2016), stimulation in DG activated the target CA3 when a high number of axons were active in the tunnels. In turn, these axons from stimulation in DG produced a surprisingly sparse activation of electrodes in CA3. While total DG source activity strongly correlated with axon activity, interestingly, total neural activity in the target CA3 failed to correlate with tunnel activity. This uncorrelated CA3 activity suggested that rate coding was not highly distributed in CA3, but rather that a more specific code, possibly sparse, governed the CA3 response. We note that a code proportional to aggregate firing rates would propagate a strong burst-like signal to a subsequent circuit, which would be less amenable to discriminable inference. In the case of CA3 as source, axon tunnel activity was proportional to the DG response, perhaps partly from re-excitation from DG; the weaker correlation of CA3 with tunnel axon activity confirmed that aggregate CA3 activity was largely independent of axonal inputs and less effective than DG at driving the activity in the tunnels, both of which were consistent with hippocampal anatomy.

Coding can be highly distributed or sparsely distributed. The Kurtosis measure supported the hypothesis that the coding was sparse in the transfer of information from DG to CA3; intuitively this measure is large when one target electrode is highly active for one stimulation source but quiet otherwise. We found a kurtosis index much smaller in DG when CA3 was stimulated than the reverse case, confirming the observation that stimulation at each site in DG evoked responses in DG, that were then transmitted through the tunnels, finally activating a relatively small set of unique responses in CA3; hence the observation that the code was sparsely distributed in CA3 population during stimulation in DG. Among the overall active neurons, only a small fraction is active following stimulation at any particular stimulation site, which may reflect sparse connectivity reported by others *in vivo* (Guzman et al., 2016). Perhaps each active CA3 neuron is functionally connected to a limited number of other active neurons not only in CA3 but also in DG. The study of the relationship between structural and functional connectivity (Poli et al., 2016) in these co-cultured DG-CA3 networks is an interesting point for a new work.

In this paper we further addressed the question about how specific the sparse population activity in CA3 was for each information source in DG. By using all target electrodes with the three highest spike rates (approximately the three most active electrodes for each stimulation site; 14%), we optimally identified 64% of specificity of responses in CA3 evoked by each stimulation site in DG.

Since the number of spikes in the post-stimulus spiking activity could be useful to estimate the selectivity of the evoked responses to the stimulation sites (Tessadori et al., 2012; Pimashkin et al., 2016), we also implemented a frequency rate-based approach to analyze the repeatability of spike rates among 25 stimulus trials. By means of learning algorithms for classification (SVM) we investigated the decoding schemes (DeMarse et al., 2001; Cozzi et al., 2005; Novellino et al., 2007; Doud et al., 2011; Pimashkin et al., 2016) in each sub-region of our engineered networks. We found that CA3 decoded the stimulation sites in DG in 43% of the trials, showing percentages correct significantly higher than controls which were significantly less reliable in decoding for the source of stimulus. These results suggest significant potential schemes which would comprise a part of how information is transmitted within our co-cultured networks, with possible import for structures in the brain.

Therefore, our experiments showed that it is possible to infer the identity of specific DG stimulation sites from evoked responses of CA3 co-cultured neurons. Further, this inference was consistent with the theory of sparse coding (Kanerva, 1988; Willomore et al., 2000), where a small number of neurons were selectively active (or not active) for a particular stimulus site; this was supported not only by an optimum of uniqueness of 64% but also by the kurtosis measure as evidence of sparse coding (Section Kurtosis Measure).

The behavior of our cultured networks likely depends on some of the anatomical relationships described *in vivo* for the DG, hilus and CA3. *In vivo* studies have shown that DG granule cells form distinctive unmyelinated axons that project along the mossy fiber pathway to CA3. Before reaching the CA3 pyramidal cells, the granule cells synapse with DG mossy cells and GABAergic interneurons (Amaral et al., 2007), innervating more inhibitory than excitatory cells (Acsády et al., 1998) with possible “back-projection” to dentate gyrus (Scharfman, 2007). This anatomical feature of the hippocampus supports what was found by Guzman et al. (2016), i.e., that increased activity of granule cells suppresses the overall excitability of the CA3 pyramidal cells, regulating the glutamate release in the mossy fiber system. This finding could explain the low release probability in the mossy fiber synapses (Lawrence et al., 2004; Vyleta and Jonas, 2014) and, therefore, the low and sparse population activity in our CA3 co-cultures. Furthermore, though there is growing evidence for a still unclear co-localization of GABAergic and glutamatergic neurotransmitters within mossy fiber terminals (Sandler and Smith, 1991), the granule cell synapses in DG tend to be glutamatergic (i.e., excitatory). This well-known feature of the hippocampal anatomy could further explain the significantly higher spike rates in DG cultures than those observed in the CA3 region, especially during electrical stimulation.

However, our two-dimensional DG-CA3 paired networks have limitations for modeling the three-dimensional network of the *in vivo* hippocampal network. Our networks lack inputs from the entorhinal cortex (EC) into DG as well as the perforant path into CA3 and other external modulatory inputs. Nevertheless, the rich repertoire and coding specificity that emerges from intrinsic neuron properties in our engineered networks is surprising given the absence of chronic behavioral stimulation during development. This activity is higher than often seen in hippocampal slices and *in vivo* models (Jung and McNaughton, 1993; Leutgeb et al., 2007). Finally, our active sparse and specific code in DG-CA3 networks with the other subregion pairs undoubtedly depend on differences in the passive properties (i.e., resting membrane potential, input resistance and membrane time constant) of the hippocampal neurons in each subregion (Kowalski et al., 2016). Their distinct proprieties of synaptic integration and input-output transformation also indicate cell type-specific evoked firing rates of these hippocampal neurons subtypes.

Therefore, our findings encourage further analysis of the contribution to information processing and transmission processes among the cultured hippocampal neurons of the other sub-regions.

## Ethics statement

This work with rats was approved by the Institutional Animal Care and Use Committee of the University of California Irvine.

## Author contributions

All authors designed the experiments. ST and DP removed stimulation artifacts from original recordings and performed spikes detection. DP analyzed spike rates, sparseness and uniqueness measures. TD implemented learning algorithms for classification (SVM). DP, GB, TD, and BW contributed to interpretation. DP wrote the article. DP, GB, BW, and TD revised the manuscript. GB supervised the study.

## Funding

This work was supported in part by NIH Grant R01 NS052233.

### Conflict of interest statement

The authors declare that the research was conducted in the absence of any commercial or financial relationships that could be construed as a potential conflict of interest.
